# The relationship between COVID-19 and hyperglycemia: screening and monitoring hospitalized patients

**DOI:** 10.1186/s40842-024-00184-7

**Published:** 2024-08-31

**Authors:** Faranak Salajegheh, Somayyeh Salajegheh, Mohsen Nakhaie, Niloofar Farsiu, Seyedeh Mahdieh Khoshnazar, Reza Sinaei, Mehrdad Farrokhnia, Soheila Torabiyan

**Affiliations:** 1 Endocrinology and Metabolism Research Center, Kerman University of Medical Sciences, Kerman, Iran; 2 Clinical Research Development Unit, Afzalipour Hospital, Kerman University of Medical Sciences, Kerman, Iran; 3 Physiology Research Center, Institute of Neuropharmacology, Kerman University of Medical Sciences, Kerman, Iran; 4 Gastroenterology and Hepatology Research Center, Institute of Basic and Clinical Physiology Sciences, Kerman University of Medical Sciences, Kerman, Iran; 5 Research Center of Tropical and Infectious Diseases, Kerman University of Medical Sciences, Kerman, Iran

**Keywords:** COVID-19, Hyperglycemia, New-onset diabetes, Prognosis

## Abstract

**Background:**

Elevated blood glucose concentration, also known as hyperglycemia, has been identified as a significant factor influencing the prognosis of COVID-19, alongside the impact of the SARS-CoV-2 infection itself.

**Methods:**

This research is a cross-sectional investigation that examined the relationship between COVID-19 and hyperglycemia in patients admitted to Afzalipour Hospital in Kerman, Iran, from July to September 2021. A standardized data sheet was used to capture demographic data (age, gender) and laboratory information (blood sugar, arterial blood oxygen saturation, and C-reactive protein (CRP)) upon admission.

**Results:**

The present research evaluated a total of 300 individuals diagnosed with COVID-19, with an average age of 50.19 ± 15.55 years. Among these patients, the majority were male, accounting for 51.67% of the total. Hyperglycemia was seen in 21.67% of patients, but less than 20% had new-onset diabetes. Individuals exhibiting hyperglycemia were typical of advanced age (*P* < 0.001). Furthermore, there was a slight but statistically significant association between advanced age and elevated blood glucose concentration (*R* = 0.254, *P* < 0.001). Gender had no significant impact on the occurrence of hyperglycemia (*P* = 0.199). There was no significant association between CRP levels and blood glucose concentration (*P* = 0.524) or the incidence of hyperglycemia (*P* = 0.473). Although there was no significant disparity in blood oxygen saturation between individuals with or without hyperglycemia (*P* = 0.06), higher blood glucose concentration was correlated with lower blood oxygen saturation (*R* = -0.151, *P* < 0.001).

**Conclusion:**

Considering the correlation between blood glucose concentration, advanced age, and disease severity, it is recommended to carefully screen and monitor all COVID-19 patients for hyperglycemia and new-onset diabetes. Effective management of these complications could enhance the control of patients’ overall prognosis and subsequent complications.

## Background

The COVID-19 outbreak in Wuhan originated on December 12, 2019, with patients displaying similar clinical signs, including fever, cough, shortness of breath, and distinctive pneumonia [1]. The first instance of this event outside of China was reported on January 13 in Thailand. As the disease progressively invaded various countries, the World Health Organization (WHO) declared a global health emergency on January 30, and COVID-19 was officially designated a pandemic on March 11, 2020 [2].

The symptoms of COVID-19 exhibit significant variability depending on the specific variants of SARS-CoV-2, the demographic characteristics of the patients, and the existence of underlying health issues [4–6]. Although many symptoms of this condition are associated with respiratory tract infections, such as fever and cough, there have also been reports of other systemic symptoms, including gastrointestinal and neurological manifestations [7]. Additional severe consequences, such as acute respiratory distress syndrome (ARDS) and acute cardiac damage, were seen in more susceptible cases [8]. Several lingering symptoms, including fatigue and shortness of breath, were noted in afflicted individuals until weeks after the infection [8, 9].

Some studies have indicated that the viral load of SARS-CoV-2 is notably higher among individuals with diabetes [10–12]. Both pre-existing diabetes and new-onset hyperglycemia are identified as crucial factors influencing the prognosis of COVID-19 [10, 11, 13]. Importantly, several investigations have documented instances of COVID-19 individuals exhibiting elevated blood glucose concentrations, irrespective of a prior diabetes diagnosis [14].

Corticosteroid medications, inflammation, stress hormones, and antibiotic treatments are a few of the common causes of hospitalization-related insulin resistance (IR) [15]. Elevated cytokine levels in COVID-19 individuals may exacerbate IR, leading to elevated blood sugar levels and decreased insulin production. Furthermore, SARS-CoV-2 can infiltrate endocrine pancreatic cells, compromising beta cell insulin release and exacerbating pre-existing diabetes. The mentioned beta-cell dysfunctions may result in diabetic ketoacidosis, hyperglycemia, and potentially new diabetes cases [16, 17].

Hyperglycemia during a COVID-19 infection, especially in severe cases, can be dangerous. For example, it can cause a sudden rise in inflammatory factors because of the higher blood glucose level and the activation of ACE2 receptors, which lets SARS-CoV-2 attach to these receptors [20]. The elevated interaction between aberrantly glycosylated ACE2 in uncontrolled hyperglycemia and the virus contributes to increased viral replication, potentially leading to greater disease severity [21].

Although several studies have examined the frequency of hyperglycemia in COVID-19 patients, especially those with pre-existing diabetes mellitus, there is still a critical need for research that explicitly targets people who do not have a previous diagnosis of diabetes. This research specifically targeted people who did not have pre-existing diabetes, a group that has not been extensively studied in the current literature. Our objective was to shed light on COVID-19-associated hyperglycemia by examining the prevalence and correlates of this subgroup’s hyperglycemia and new-onset diabetes. Additionally, our research was conducted at Afzalipour Hospital in Kerman, Iran, providing vital data from a region that may have distinctive patterns of illness occurrence and treatment. By collecting and analyzing the data thoroughly, we aimed to provide new insights that improved our comprehension of the intricate connection between COVID-19 and blood sugar problems. The results of the study will help develop more efficient approaches to patient treatment and care.

## Methods

### Study population

This cross-sectional retrospective study was focused on COVID-19 patients hospitalized at Afzalipour Hospital in Kerman, Iran, from July to September 2021, who were discharged in a healthy or stable condition. The patients’ data were obtained from hospital records, and the study population was selected according to the inclusion and exclusion criteria described later.

### Inclusion and exclusion criteria

The inclusion criteria for the study included:

- Positive RT-PCR test result for the SARS-CoV-2 virus or a chest computed tomography (CT) scan indicating evidence in favor of infection with this virus;
- Patient’s full consciousness;
- Age 20 ≤ and ≥ 50 years (excluding age ranges with a high risk of diabetes 1 and 2 development).

The exclusion criteria included:

- Previous definite diabetes 1 or 2 (measured by fasting blood sugar (FBS) and HbA1c) or glucose tolerance disorders;
- Pregnancy (due to the risk of gestational diabetes);
- Use of corticosteroid drugs (which can lead to hyperglycemia);
- Lack of patient file information;
- Occurrence of death during hospitalization.

**Fig. 1:**
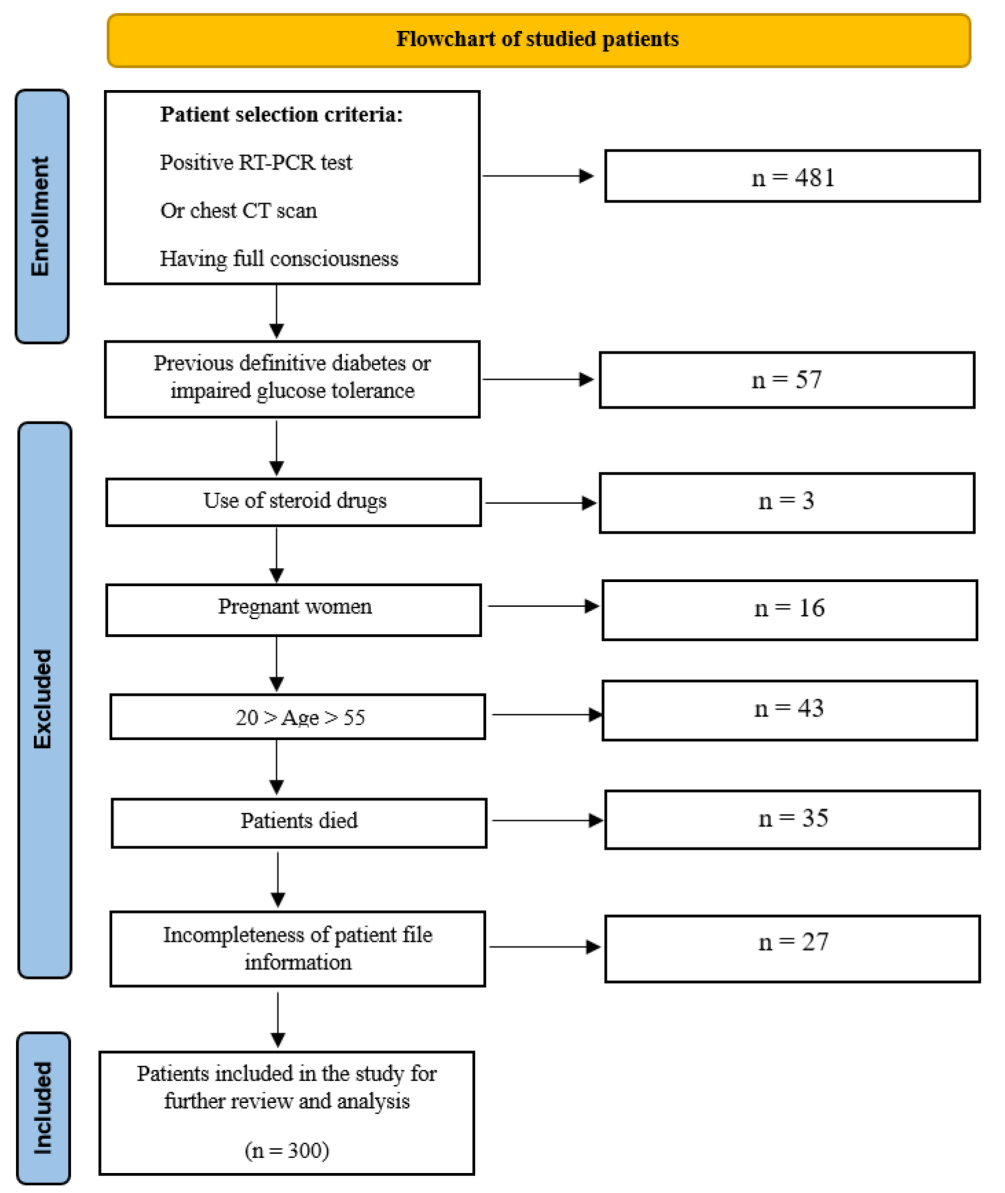
Flowchart for the method of enrolling the study population

The methodology for enrolling the study population is summarized in the flowchart provided in Fig. 1.

### Data collection

After the patients were selected, relevant information from the patients’ records was collected using a comprehensive data sheet. The collected data included demographic details such as age and gender, as well as laboratory information such as blood glucose concentration, arterial blood oxygen saturation (SpO2), and C-reactive protein (CRP) serum levels upon admission.

Specific criteria were established to identify hyperglycemia. Patients were considered to have hyperglycemia if they exhibited at least one of the following on the first day of admission:

- Two-hour postprandial glucose (2hpp) ≥ 200 mg/dL.
- FBS ≥ 126 mg/dL.

It should be noted that glucose tolerance disorders, dysglycemia that comprises both prediabetes and diabetes, including conditions of impaired fasting glucose (IFG), impaired glucose tolerance (IGT), and diabetes mellitus (DM), were also recognized with FBS between 110 and 125 mg/dL or 2hpp 140–199 mg/dL.

### Statistical analysis

The collected data were analyzed using SPSS statistical software (version 27). Descriptive statistics, such as central tendency indices (mean and standard deviation), as well as frequency and percentage, were used to categorize and summarize the findings. The chi-squared test, student’s t-test, and Pearson correlation coefficient (R) were used to investigate the relationship between COVID-19 and demographic variables with hyperglycemia. Meanwhile, *P.values* less than 0.05 were considered significant.

## Results

### Demographic and clinical information

The research included 300 patients diagnosed with COVID-19. The average age was 50.19 ± 15.55 years, with a majority of men (51.67%), as shown in Table 1. Hyperglycemia was identified in 65 patients (21.67%), and new-onset diabetes was diagnosed in 17.67% of cases (Table 1). In addition, the average blood glucose concentration, blood oxygen saturation, and CRP levels were also measured among all patients (Table 1).

**Table 1:** Distribution of investigated variables in the study in patients with COVID-19. Data on parameters are expressed either as absolute numbers and percentage values or as mean ± standard deviation. Patients were considered to have hyperglycemia if they exhibited two-hour postprandial glucose (2hpp) ≥ 200 mg/dL or fasting blood sugar (FBS) ≥ 126 mg/dL on the first day of admission. CRP: C reactive protein

Parameters	Data
Age (years)	50.19 ± 15.55
Gender (male, number (%))	155 (51.67)
Gender (female, number (%))	145 (48.33)
Hyperglycemia (number (%))	65 (21.67)
New onset diabetes (number (%))	53 (17.67)
Blood glucose concentration (mg/dL)	156.83 ± 89.8
Blood oxygen saturation (%)	87.03 ± 6.64
CRP (mg/dL)	43.61 ± 30.36

### Relationship between hyperglycemia, new onset of diabetes, and gender

There was no significant difference in gender distribution between patients with or without hyperglycemia (*P* = 0.199). However, patients with hyperglycemia had a significantly higher rate of new-onset diabetes diagnosis (*P* < 0.001) (Table 2).

**Table 2:** Gender distribution and new onset of diabetes in patients with COVID-19 with or without hyperglycemia. Patients were considered to have hyperglycemia if they exhibited two-hour postprandial glucose (2hpp) ≥ 200 mg/dL or fasting blood sugar (FBS) ≥ 126 mg/dL on the first day of admission

Parameters	Hyperglycemia (Number, (%))	No hyperglycemia (Number, (%))	*P*.value
Gender (male)	29 (44.62)	126 (53.62)	0.199
New onset diabetes	33(50.77)	20 (8.51)	< 0.001

### Relationship of hyperglycemia with quantitative parameters

Patients with hyperglycemia exhibited a higher age (*P* < 0.001) and higher blood glucose concentration (*P* < 0.001) compared to others. No significant differences were observed in blood oxygen saturation (*P* = 0.06) and serum CRP (*P* = 0.473) between the two groups (Table 3).

**Table 3:** Distribution of age, blood oxygen saturation, C reactive protein (CRP), and blood glucose concentration in patients with COVID-19 with or without hyperglycemia. The patients were considered to have hyperglycemia if they exhibited two-hour postprandial glucose (2hpp) ≥ 200 mg/dL or fasting blood sugar (FBS) ≥ 126 mg/dL on the first day of admission. SD: standard deviation; CRP: C reactive protein

Parameters	Hyperglycemia (Mean ± SD)	No hyperglycemia (Mean ± SD)	*P*.value
Age (year)	56.46 ± 12.7	48.5 ± 15.84	< 0.001
Blood oxygen saturation (%)	85.66 ± 7.85	87.41 ± 6.23	0.06
CRP (mg/dL)	46.23 ± 34.01	42.89 ± 29.31	0.473
Blood glucose concentration (mg/dL)	300.03 ± 85.04	117.22 ± 32.47	< 0.001

### Correlation analysis

Examining correlations between quantitative variables revealed that CRP levels were not significantly related to blood glucose concentration (*P* = 0.524). However, age showed a slight yet positive significant relationship with blood glucose concentration (*R* = 0.254, *P* < 0.001), while blood oxygen saturation exhibited a slight yet negative significant relationship with blood glucose concentration (*R* = -0.151, *P* < 0.001) (Table 4).

**Table 4:** Correlation between age, C reactive protein (CRP), and blood oxygen saturation with blood glucose concentration in study patients. CRP: C reactive protein. ** *P* < 0.01

Parameters		Blood glucose concentration (mg/dL)
Age (year)	Correlation coefficient®	0.254**
	*P.value*	< 0.001
CRP (mg/dL)	Correlation coefficient®	0.037
	*P.value*	0.524
Blood oxygen saturation (%)	Correlation coefficient®	-0.151**
	*P.value*	0.009

## Discussion

Multiple studies have shown that the presence of high levels of blood glucose concentration and hyperglycemia are not only influenced by the SARS-CoV-2 infection but also play a role in defining the severity and eventual outcome of COVID-19. Our study was designed to examine the links between COVID-19 and hyperglycemia, focusing on individuals without prior diabetes diagnosis. The objective was to determine whether COVID-19-induced hyperglycemia could contribute to the development of new-onset diabetes. This experiment focused on patients in Kerman, Iran, and aimed to determine whether regional disparities provide distinct results compared to other investigations.

The findings of our study revealed that over 20% of hospitalized COVID-19 patients experienced hyperglycemia, with less than 20% developing new-onset diabetes. In terms of demographic factors, advanced age correlated with both the presence of hyperglycemia and higher blood glucose concentration, while gender did not significantly influence the occurrence of hyperglycemia. Additionally, CRP levels did not impact blood glucose concentration or the occurrence of hyperglycemia. Although there was no significant difference in blood oxygen saturation between patients with or without hyperglycemia, lower blood oxygen saturation levels were associated with elevated blood glucose concentration.

In our study, the incidence of hyperglycemia and new-onset diabetes was found to be 21.67% and 17.67%, respectively. This rate of hyperglycemia occurrence in COVID-19 patients without a previous history of diabetes varied across similar studies, ranging from 4% to more than 20% [22–26]. Unfortunately, the patients in our study are on the higher end of the spectrum. It is difficult to accurately determine the occurrence of hyperglycemia and new-onset diabetes in COVID-19 patients since it is challenging to distinguish between cases where individuals had pre-existing high blood glucose concentrations that remained undetected before developing COVID-19 and cases in which hyperglycemia developed after the infection.

The present study emphasizes the need for further research to carefully assess COVID-19 patients for prior elevated blood glucose concentrations in order to acquire more dependable findings, with a particular focus on the occurrence of new-onset diabetes. It is essential to additionally take into account confounding variables, such as the treatment plans for COVID-19, particularly corticosteroids, and the influence of IR during severe diseases like sepsis on high blood glucose concentrations [18, 19, 27].

Our study revealed that aging is associated with a higher risk of hyperglycemia in patients with COVID-19, and there is a positive correlation between age and blood glucose concentrations. This finding aligns with the results of previous similar studies, highlighting that elderly patients are at a greater risk for hyperglycemia [28–30]. This association may be attributed to the weaker immune response and increased susceptibility to infections in older individuals, leading to more severe clinical symptoms following the SARS-CoV-2 infection [31].

Also, our study found that gender did not have a significant impact on the occurrence of hyperglycemia in patients with COVID-19. While this study did not specifically investigate the link between gender and the occurrence of hyperglycemia in patients with COVID-19, the absence of a significant relationship between the two parameters was expected. This is because previous research has shown that men are more likely to experience severe COVID-19 due to cultural, social, and behavioral factors that increase their exposure to the virus. Conversely, it has been shown that women often have a more robust immune response to illnesses and immunizations, leading to potentially severe consequences. Gender-related disparities may have mitigated the influence of gender on the prevalence of hyperglycemia in the setting of COVID-19 [32].

When comparing individuals with or without hyperglycemia, there was no significant difference in CRP levels or blood oxygen saturation, which are both indicators of disease severity and inflammation. However, lower blood oxygen levels are often correlated with higher blood glucose concentrations. The etiopathological role of inflammation in the development and progression of hyperglycemia and type 2 diabetes was previously recognized. Inflammatory cytokines can induce structural and functional abnormalities in endothelial cells, leading to impaired insulin secretion, damage to pancreatic beta-cells, and, ultimately, an increase in blood glucose concentration [33–36]. Hence, the presence of increased inflammation after acquiring SARS-CoV-2 and IR is to be anticipated. Nevertheless, our findings do not provide definitive proof of a direct correlation between hyperglycemia and these inflammatory markers. It is recommended that future studies investigate the incidence of mild COVID-19 cases (outpatient cases) and the likelihood of hyperglycemia to further elucidate the relationship between COVID-19, inflammation, and hyperglycemia.

In addition to infection, medications, and inflammation, stress can also contribute to hyperglycemia and lead to the development of new-onset diabetes. Future studies can explore the stress hyperglycemia ratio (SHR), which measures the ratio of blood glucose (mmol/L) per HbA1c (%) [27]. The evaluation of SHR can provide valuable information about the progression and severity of the disease. By incorporating the SHR into future research, our comprehension of stress-induced hyperglycemia in COVID-19 patients can be improved. This will provide a thorough evaluation of stress-induced hyperglycemia’s effect on the prognosis of the disease.

The current study has several limitations, including its retrospective nature, the examination of COVID-19 patients in a single medical facility, and a lack of investigation into the relationship between blood glucose concentration, hyperglycemia, and the course and prognosis of the disease. Furthermore, the research failed to do post-recovery and discharge follow-ups on blood glucose concentration, particularly in the long term. In addition, the study did not gather any data on the symptoms reported by the participants, which could have supplied useful information about how COVID-19 presents clinically in relation to hyperglycemia. Furthermore, the study did not distinguish between different variants of the SARS-CoV-2 virus, which could have given invaluable perspectives into the virus’s attributes and its potential effects on hyperglycemia. Future studies are recommended to overcome these limitations and provide a more comprehensive understanding of the topic. Despite these limitations, the study’s strengths lie in its substantial sample size and the examination of crucial factors influencing disease severity, as well as their interplay in the occurrence of hyperglycemia and blood glucose concentration.

In conclusion, given the significant occurrence of hyperglycemia and new-onset diabetes in COVID-19 patients, particularly among seniors, and the observed association between blood glucose concentration and increased disease severity, it is crucial to screen and closely monitor all COVID-19 patients for hyperglycemia and new-onset diabetes. This proactive approach is essential for controlling the relative prognosis of these patients and mitigating subsequent complications through appropriate management of this complication.

## Data Availability

The datasets of the present investigation can be obtained from the corresponding author following an adequate request.
